# Volar Locking Plate Versus External Fixation for Distal Radius Fractures: A Systematic Review and Meta-Analysis of Randomized Controlled Trials

**DOI:** 10.7759/cureus.100789

**Published:** 2026-01-04

**Authors:** Mohamed Zahed, Mahmoud Elmesalmi, Ziad El Menawy, Nour Elnaggar, Farouk Ahmed, Mahmoud Odeh, Rawad Azaz, Sara E Elbahnasawy, Hussein M Elgendy, Mohamed Hesham Gamal

**Affiliations:** 1 Orthopaedics, John Radcliffe Hospital, Oxford University Hospitals NHS Trust, Oxford, GBR; 2 Trauma and Orthopaedics, St George's University Hospitals, London, GBR; 3 Trauma and Orthopaedics, University Hospital of Wales, Cardiff, GBR; 4 Faculty of Medicine, Zagazig University, Zagazig, EGY; 5 Emergency Department, Queen Alexandra Hospital, Portsmouth, GBR; 6 Trauma and Orthopaedics, Cardiff and Vale University Health Board, Cardiff, GBR; 7 Trauma and Orthopaedics, London Royal Free NHS Trust, London, GBR; 8 Diagnostic Radiology, Menofia University Hospital, Menofia, EGY; 9 General Practice, Misr University for Science and Technology, Giza, EGY; 10 Pharmacology and Therapeutics, Faculty of Pharmacy, Tanta university, Tanta, EGY

**Keywords:** dynamic external fixation, fracture of distal radius, pain management, randomized trials, systematic review and meta analysis, volar locking plate

## Abstract

Distal radius fractures (DRFs) are among the most common fractures of the upper extremity, and management remains controversial. Both volar locking plate (VLP) and external fixation (EF) are widely used. We aim to compare functional, radiographic, and safety outcomes between VLP and EF in the treatment of DRFs to guide evidence-based management. We systematically searched PubMed, Scopus, Web of Science, and Embase through October 2025 for English-language RCTs comparing VLPs versus EFs in adults with DRFs. Quality assessment was performed using the Cochrane Risk of Bias tool (version 2). Meta-analysis was conducted using RevMan 5.4. Heterogeneity was assessed using I² statistics, and random-effects models were applied when I² exceeded 50%. Nine randomized controlled trials (RCTs) involving 1,023 patients were included. VLP showed better early functional recovery, with significantly lower Disabilities of the Arm, Shoulder, and Hand (DASH) scores at month 1 (mean difference (MD) = −7.94, p = 0.009) and month 6 (MD = −4.26, p = 0.010). Grip strength favored VLP at 3 months (MD = 5.35, p = 0.002), 6 months (MD = 5.30, p = 0.002), and 1 year (MD = 2.96, p = 0.005). Wrist range of motion (ROM) also favored VLP across all time points, with improvements in MD of 4.18 degrees over 3 months and 2.73 degrees over 12 months. Radiographic evaluation indicated that VLP achieved greater volar tilt (MD = 1.84, p = 0.02), whereas EF resulted in better ulnar deviation (MD = −0.82, p = 0.001). VLP had a significantly higher risk of reoperation (RR = 2.51, p < 0.0001). VLP fixation provides earlier functional recovery and better wrist mobility than EF in the management of DRFs. EF, however, is associated with a lower overall risk of reoperation. Although long-term functional outcomes and radiographic parameters are comparable between the two techniques, VLP offers clear short-term advantages, supporting its use when early functional restoration is a priority.

## Introduction and background

Distal radius fractures (DRFs) are the most prevalent fractures in the upper extremity, accounting for 25% of pediatric and up to 18% of elderly fractures. Although most frequently in children and older adults, they also significantly affect young adults [1,2]. The management of DRFs remains controversial, with treatment options ranging from non-operative approaches to various forms of external and internal fixation [3]. Proper anatomical alignment during fixation is essential to restore function and optimize outcomes. However, malunion can occur, potentially leading to post-traumatic osteoarthritis and long-term functional impairment [4].

While stable DRFs can be treated non-operatively with acceptable outcomes [5], a substantial proportion of patients with unstable DRFs still require surgical intervention for proper alignment [6]. Among surgical options, external fixation (EF) has been traditionally used, but volar locking plate (VLP) fixation has gained increasing popularity in recent years [7]. The volar approach offers stable fixation while minimizing the risk of dorsal extensor tendon injury. Nevertheless, complications associated with VLP fixation remain a concern [8]. A 2023 systematic review by Chinemerem Nwosu et al. reported an overall complication rate of approximately 30%. The median nerve-related issues (7.1%) and hardware removal (6.8%) were the most frequently reported. Additionally, tenosynovitis represented the most common tendon-related complication, occurring in 3.4% of cases [9].

The current evidence is limited, as the Cochrane Collaboration reported that the best treatment approach for DRFs remains controversial [10]. As a result of this debate, we aim to compare VLPs and EFs with respect to functional outcomes, radiographic parameters, and complication rates in the management of DRFs to support evidence-based treatment decisions.

## Review

Methods

We performed this systematic review and meta-analysis following the Cochrane handbook and the Preferred Reporting Items for Systematic Reviews and Meta-Analyses (PRISMA) guidelines [11,12].

Search Strategy

We systematically searched PubMed, Scopus, Web of Science, and Embase for relevant studies published up to 25 October 2025. In our search strategy, we used the following terms: "Radius" OR "Radius Fractures" OR "Wrist Fractures" and detailed search as: (distal radius OR distal radial OR smith) AND (fractur* OR break* OR injury) ) AND ( "Bone Plates" OR "Internal Fixators" OR (volar OR palmar) AND (plate* OR ORIF) OR "locking plate*" OR "locking compression plate*" OR LCP OR VLP OR "internal fixat*" ) AND ( "External Fixators" OR "external fixat*" OR "dynamic external" OR "non-bridging" OR "bridging external" OR "hoffmann II" OR "unilateral frame" ). The details of the complete search strategy are provided in Appendix A.

Study Selection and Eligibility Criteria

We included only English-language randomized controlled trials (RCTs) that fulfilled the following eligibility criteria: (1) population: Adults with DRFs, (2): intervention: Surgery using a volar locking plate (VLP) (internal fixator on the palm side),(3) comparator: Surgery using an external fixator (a frame outside the skin), and (4) outcomes: Primary outcomes: patient-reported hand and wrist function-secondary outcomes: complication rate, range of motion (ROM), rate of secondary surgery, and radiographic alignment.

Data Extraction

Data were extracted using predesigned Excel sheets to collect information into two main categories: (1) Study summary data, including the Study ID, country, sample size, intervention details, disease type, time from injury to surgery, follow-up duration, and primary outcomes. (2) Baseline characteristics, including Study ID, study groups (n), mean age (SD), sex distribution (male, n (%)), injury in the dominant hand (n (%)), injury mechanism (high-energy, n (%)), AO fracture classification (C1, C2, and C3, n (%)), and the number of cases that underwent closed reduction before surgery (n (%)).

Quality Assessment

The quality of the included RCTs was evaluated using Cochrane’s Risk of Bias Tool (version 2) [13]. Five domains: randomization process (D1), deviations from intended interventions (D2), missing outcome data (D3), measurement of outcomes (D4), and selection of reported results (D5). Each study was rated as low risk, some concerns, or high risk of bias.

Data Analysis

Statistical analyses were conducted using RevMan version 5.4 with the significance level set at p < 0.05. For continuous data, the mean difference (MD) and 95% confidence interval (CI) were calculated; for dichotomous data, the risk ratio (RR) was calculated. Heterogeneity among studies was evaluated using both the I² statistic and the chi-square test. Data were considered heterogeneous when the chi-square test yielded a p-value < 0.1, and the I² statistic was >50%. A fixed-effect model was employed for homogeneous data, while a random-effects model was utilized in cases of significant heterogeneity.

Outcome Measures

Functional disability was assessed using the Disabilities of the Arm, Shoulder, and Hand (DASH) score or Quick DASH (QuickDASH) questionnaire. The DASH is a 30-item validated instrument, while QuickDASH is an abbreviated 11-item version; both generate scores from 0 to 100, with higher scores indicating greater functional impairment and worse outcomes [14,15].

Pain intensity was measured using the visual analog scale (VAS), a standardized 10-cm scale ranging from 0 (no pain) to 10 (the worst imaginable pain). Lower scores indicate better pain control [16].

Wrist range of motion (ROM) was assessed using standard goniometry to measure four primary movements: flexion, extension, pronation, and supination, reported in degrees. Greater ROM values indicate better functional recovery [17].

Grip strength was measured using a hand dynamometer, with values reported in kilograms or as a percentage of the contralateral (uninjured) hand. Higher values indicate better functional recovery [18].

Radiographic parameters included volar tilt (normal: 11-12 degrees), radial inclination (normal: 22-23 degrees), ulnar deviation (standard: 0 to -1 mm), and radial shortening, measured in millimeters or degrees on standard posteroanterior and lateral radiographs. Values closer to anatomical norms indicate better fracture reduction and alignment [19].

Reoperation was defined as any unplanned secondary surgical procedure related to the initial fracture treatment, including hardware removal for symptomatic implants, management of complications, or revision surgery for malunion.

Results

Literature Search and Study Selection

**Figure 1:**
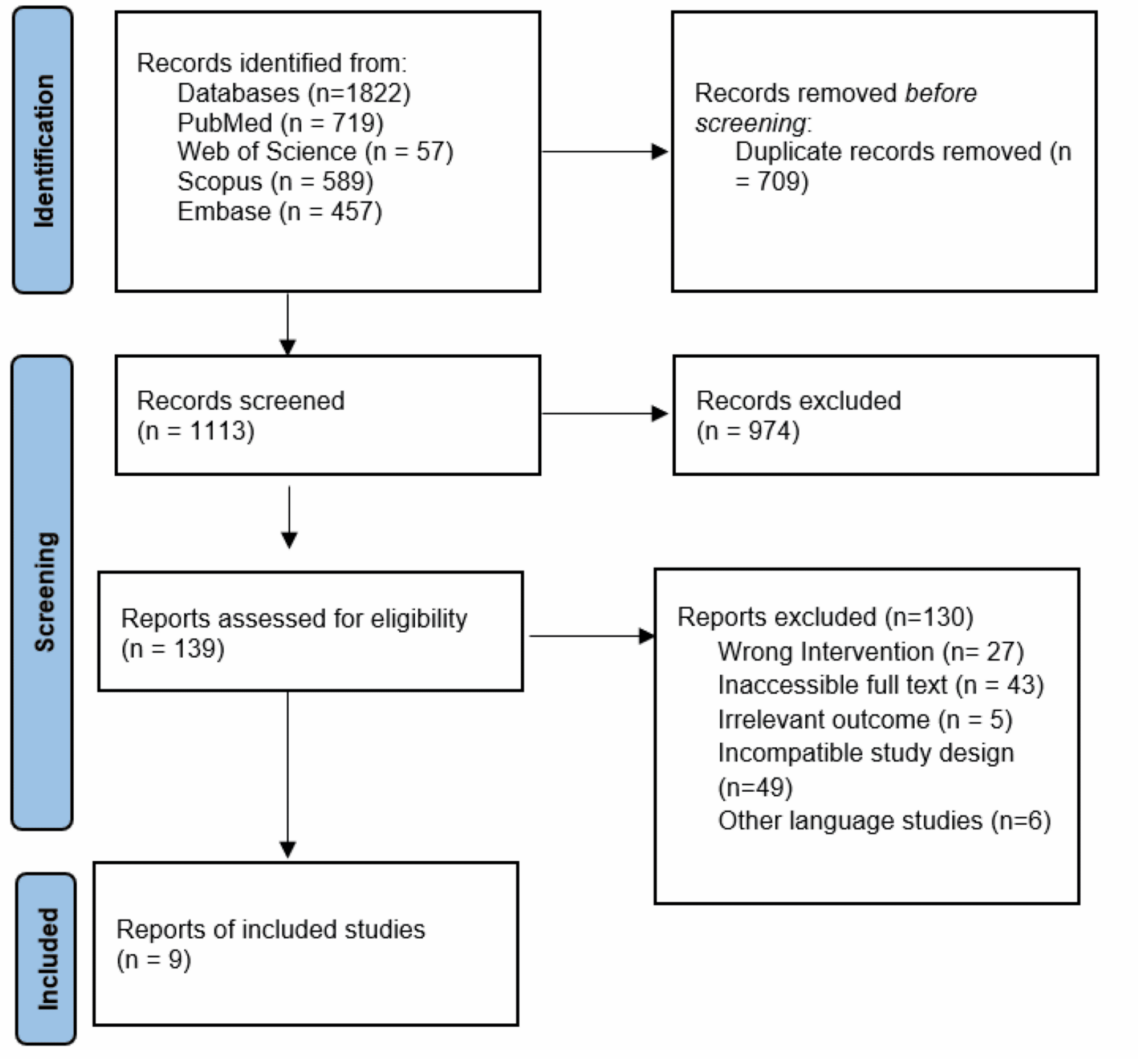
PRISMA flow diagram References: [20-28]

Based on our search strategy, we identified 1,113 studies after removing 709 duplicates. Following title and abstract screening, 139 studies were retained for full-text review. Of these, nine studies met the inclusion criteria and were included in the final analysis (Figure 1) [20-28].

Baseline Characteristics

Baseline characteristics were generally well-balanced between groups across all studies. The mean patient age ranged from 38.9 to 61 years. Male representation varied across studies, ranging from 10% to 58% of participants. Injury to the dominant hand occurred in 37% to 55% of patients. High-energy trauma mechanisms were documented in 12% to 58% of cases across four studies. Closed reduction prior to definitive surgical fixation was performed in 84% to 100% of cases where reported. Full data are in Table 1.

**Table 1:** Baseline characteristics References: [20-28] NO: number; SD: standard deviation; NR: not reported; NA: not applicable; AO: Association for the Study of Internal Fixation

Study ID	Groups (n)	Age, Mean (SD)	Sex (male), NO. (%)	Injury in the Dominant Hand, NO. (%)	Injury Mechanism (High-Energy), NO. (%)	AO Classification (C1), NO. (%)	AO Classification (C2), NO. (%)	AO Classification (C3), NO. (%)	Closed reduction prior to surgery, NO. (%)
Gupta et al., 2025 [26]	External Fixator (97)	42 (10.2)	54 (56%)	45 (46%)	54 (56%)	25 (26%)	39 (40%)	33 (34%)	97 (100%)
	Volar Plating (96)	40 (12.1)	56 (58%)	50 (52%)	56 (58%)	22 (23%)	38 (40%)	36 (37%)	96 (100%)
Hammer et al., 2019 [27]	External Fixator (82)	54 (12.4)	27 (33%)	37 (45%)	15 (18%)	3 (4%)	51 (62%)	28 (34%)	76 (93%)
	Volar Plating (84)	56 (10.5)	25 (30%)	39 (46%)	10 (12%)	5 (6%)	49 (58%)	30 (36%)	70 (84%)
Egol et al., 2008 [28]	External Fixator (44)	49.9 (Range 18-78)	22 (50%)	39 (89%)	NR	Total Type C: 26 (59%)			44 (100%)
	Volar Plating (44)	52.2 (Range 19-87)	19 (43%)	39 (89%)	NR	Total Type C: 17 (38%)			44 (100%)
Roh et al., 2015 [20]	External Fixator (38)	55.3 (11.2)	14 (36.8%)	NR	NR	0 (0)	24 (63.2%)	14 (36.8%)	NR
	Volar Plating (36)	54.4 (10.9)	16 (44.4%)	NR	NR	0 (0)	21 (58.3%)	15 (41.7%)	NR
Shukla et al., 2014 [21]	External Fixator (62)	38.9 (13.1)	29 (47%)	NR	NR	NR	NR	NR	NR
	Volar Plating (48)	39.3 (13.1)	20 (42%)	NR	NR	NR	NR	NR	NR
Ludvigsen et al., 2020 [22]	External Fixator (81)	57 (Range 20-70)	8 (10%)	30 (37%)	NR	NA	NA	NA	NR
	Volar Plating (75)	56 (Range 20-70)	8 (11%)	38 (51%)	NR	NA	NA	NA	NR
Wei et al., 2009 [23]	External Fixator (22)	55 (16)	6 (27%)	12 (55%)	NR	Intra-articular (class C): 12 (55%)			NR
	Volar Plating (12)	61 (18)	3 (25%)	6 (50%)	NR	Intra-articular (class C): 9 (75%)			NR
Williksen et al., 2013 [24]	External Fixator (59)	54 (Range 20-84)	22 (20%)	54 (48.6%)	26 (23.4%)	18 (34.6%)	18 (34.6%)	1 (1.9%)	52 (100%)
	Volar Plating (52)					30 (50.8%)	13 (22.0%)	2 (3.4%)	59 (100%)
Williksen et al., 2015 [25]	External Fixator (45)	54 (Range 20-84)	13 (14.3%)	43 (47.2%)	22 (24.2%)	17 (37.0%)	16 (34.8%)	1 (2.2%)	46 (100%)
	Volar Plating (46)					23 (51.1%)	9 (20.0%)	2 (4.4%)	45 (100%)

Study Summary Characteristics

A total of nine RCTs were included, comprising 1,023 participants. The studies were conducted in India, Norway, South Korea, and the United States, with sample sizes ranging from 46 to 202 patients per study. Follow-up duration ranged from 1 year in six studies to 5 years in one study, with intermediate assessments at 2 and 3 years in selected studies. Four studies focused exclusively on intra-articular fractures of AO-type C, while five studies included both extra-articular and intra-articular fractures. Surgical intervention was performed at a mean of 2.9 to 7 days post-injury, where reported. Full data are in Table 2.

**Table 2:** Summary of the included studies References: [20-28] NR: Not Reported; K-wires: Kirschner wires; AO: Arbeitsgemeinschaft für Osteosynthesefragen (Association for the Study of Internal Fixation); NCT: National Clinical Trial; USA: United States of America; DVR: dorsal volar radius; MHQ: Michigan Hand Outcomes Questionnaire; OTA: Orthopedic Trauma Association; LCP: locking compression plate; QuickDASH: Quick Disabilities of the Arm, Shoulder and Hand; DASH: disabilities of the arm, shoulder and hand; PRWHE: Patient-Rated Wrist/Hand Evaluation

Study ID	Protocol Number	Country	Sample Size	Intervention Details	Disease Type	Time until Surgery (Days), Mean (SD)	Follow-Up Duration	Primary Outcomes
Gupta et al., 2025 [26]	NR	1 country (India)	193	External Fixation: Bridging external fixator (wrist distractor) with supplemental K-wires. Internal Fixation: Volar locking plate via modified Henry's approach with provisional/adjuvant K-wires	AO-type C2 and C3 distal radius fractures (intra-articular)	External Fixation: 4.5 (4) Internal Fixation: 6 (4.7)	3 years	Quick Disabilities of the Arm, Shoulder, and Hand (QuickDASH) score and Jakim’s score
Hammer et al., 2019 [27]	NCT01062997	1 country (Norway)	166	External Fixation: Bridging external fixation (Hoffman Compact T2) with 2-3 supplemental K-wires. Internal Fixation: Volar locking plate (DVR; DePuy) via modified Henry approach	AO-type C1, C2, and C3 distal radius fractures (intra-articular)	External Fixation: 6.5 (4) Internal Fixation: 7 (4.7)	2 years	Quick Disabilities of the Arm, Shoulder, and Hand (QuickDASH) score
Egol et al., 2008 [28]	NR	1 country (USA)	88	External Fixation: Bridging external fixator (EBI or Stryker) with two pins in the 2nd metacarpal and two in the radial shaft, supplemented with percutaneous K-wires. Internal Fixation: Locked pre-contoured volar plate (Hand Innovations or Stryker) via Henry approach	Unstable, displaced fractures of the distal radius	NR	1 year	Disabilities of the Arm, Shoulder, and Hand (DASH score)
Roh et al., 2015 [20]	NR	1 country (Korea)	74	External Fixation: Closed or limited open reduction with a uniplanar bridging external fixator (Orthotech Multi-Fix) and supplemental percutaneous K-wires (typically three 1.6-mm K-wires). Internal Fixation: Volar locking plate (Synthes 2.4 LCP or Medartis Aptus Radius 2.5)	AO-type C1, C2, and C3 distal radius fractures (intra-articular)	External Fixation: 2.9 (1.5) Internal Fixation: 3.2 (1.3)	1 year	Disabilities of the Arm, Shoulder, and Hand by MHQ score
Shukla et al., 2014 [21]	NR	1 country (India)	110	External Fixation: Bridging external fixator (Schanz pins in the 2nd metacarpal and radius). Internal Fixation: Volar locking plate	Displaced intra-articular distal radius fracture (Cooney’s type IV)	NR	1 year	Disabilities of the Arm, Shoulder, and Hand by Green and O'Brien score
Ludvigsen et al., 2020 [22]	NCT01904084	Norway	142	External Fixation: Hoffmann Compact T2 external fixator (Stryker) using four “apex pins” (two in the radius, two in the metacarpal). Internal Fixation: DVR volar locking plate (DePuy). A dorsal splint was applied and removed within a few days	Displaced unstable extra-articular distal radial fracture (OTA/AO-type A3)	NR	1 year	Patient-Rated Wrist/Hand Evaluation (PRWHE) score and QuickDASH score
Wei et al., 2009 [23]	NR	USA	46	External Fixation: Bridging external fixator (Hoffmann II Compact) with use of intrafocal fracture pinning under fluoroscopy and supplemental Kirschner wires. Internal Fixation: Locked volar plate (EBI OptiLock). A volar splint for comfort	Unstable distal radial fracture (OTA types A3, C1, C2, C3)	NR	1 year	Disabilities of the Arm, Shoulder, and Hand (DASH) questionnaire score
Williksen et al., 2013 [24]	NR	Norway	202	External Fixation: Hoffman II (Stryker) or Synthes distal radius fixator. Pins: 2 in the second metacarpal, 2 in the radius. Adjuvant Pins: 3 extrafocal 1.8-mm Steinmann pins. Internal Fixation: Flexor carpi radialis. Plates: Acumed Acu-Lok, Synthes 2.4 LCP, Hand Innovation DVR. Post-op: Dorsal plaster orthosis for 2 weeks	Unstable distal radius fractures (AO Types A2, A3, C1, C2, C3)	NR	1 year	Disabilities of the Arm, Shoulder, and Hand QuickDASH score
Williksen et al., 2015 [25]	NCT01278745	Norway	104	External Fixation: Hoffman II (Stryker) or Synthes distal radius fixator. Pins: 2 in the second metacarpal, 2 in the radius. Adjuvant Pins: 3 extrafocal 1.8-mm Steinmann pins. Internal Fixation: Flexor carpi radialis. Plates: Acumed Acu-Lok, Synthes 2.4 LCP, Hand Innovation DVR. Post-op: Dorsal plaster orthosis for 2 weeks	Unstable distal radius fractures (AO Types A2, A3, C1, C2, C3)	NR	5 years	Disabilities of the Arm, Shoulder, and Hand QuickDASH score

The Quality of the Included Studies

**Figure 2:**
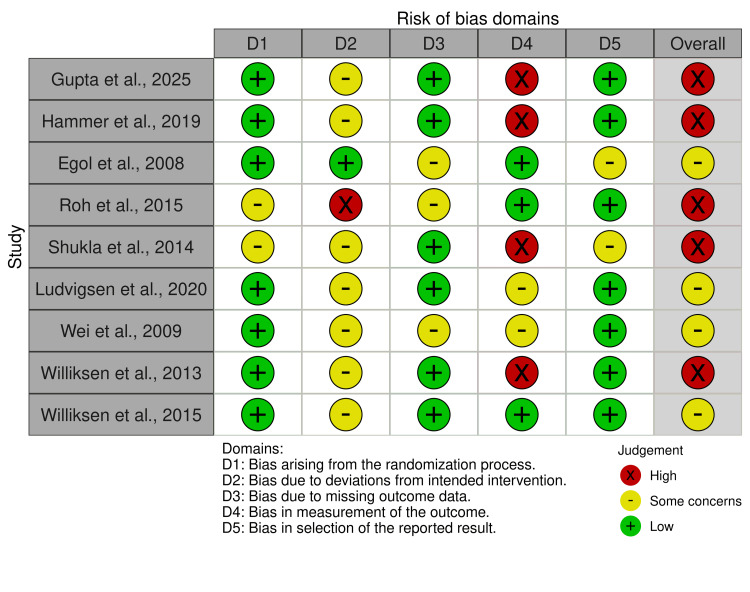
Risk of bias 2 References: [20-28]

Most of the included studies (five studies) showed a high risk of bias, mainly due to issues in Domain 4 (measurement of outcomes). This was the most common source of bias, likely resulting from the lack of blinding among outcome assessors and the subjective nature of some measured outcomes (Figure 2).

Primary outcomes

Disabilities of the Arm, Shoulder, and Hand (DASH) Score

**Figure 3:**
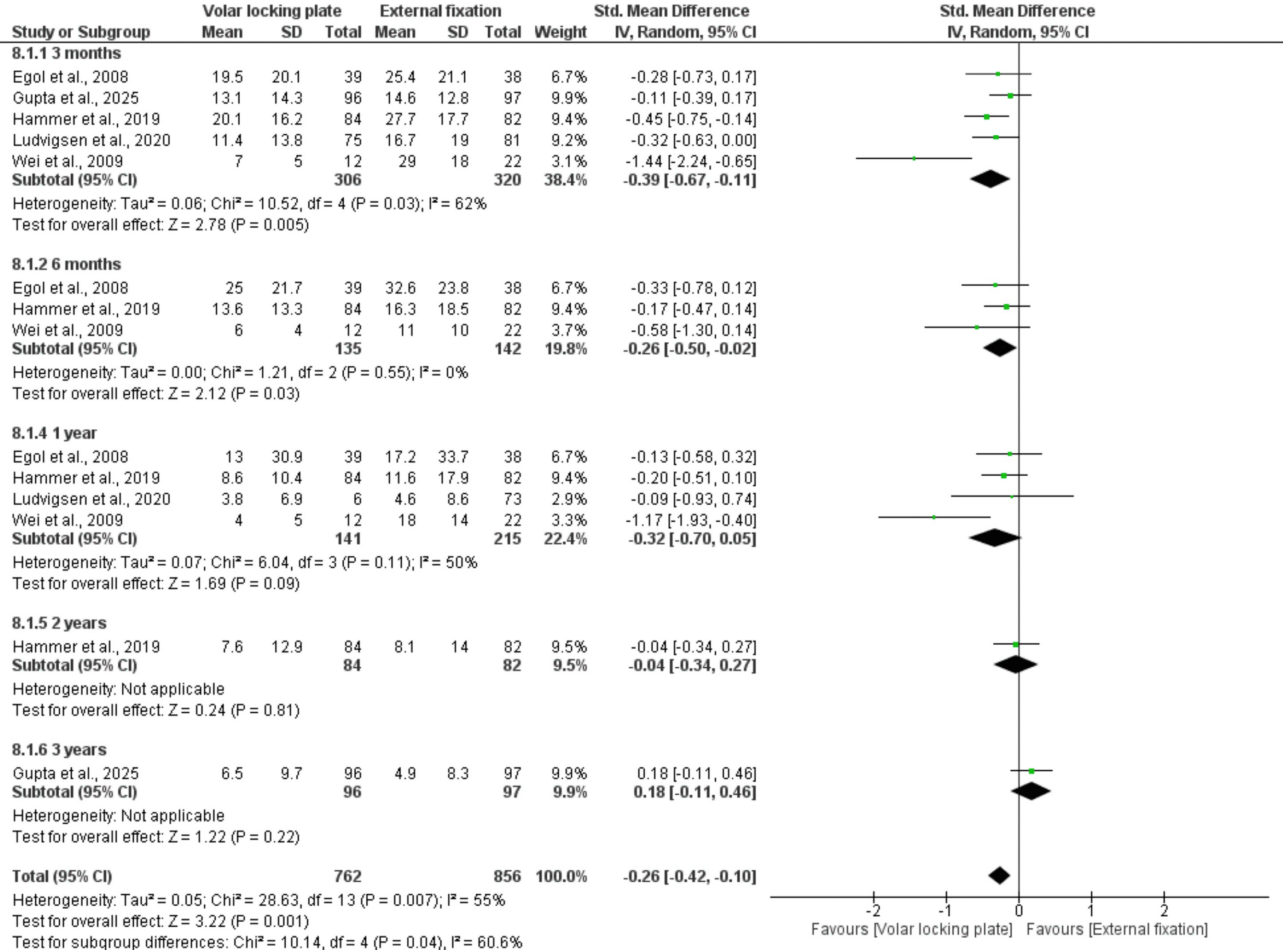
Disabilities of the Arm, Shoulder, and Hand (DASH) Score References: [22,23,26-28].

At three- and six-month post-treatment, patients managed with VLP demonstrated significantly better DASH scores, with standard mean differences of -0.39 (95% CI: −0.67 to −0.11; p = 0.005; I² = 62%) and −0.26 (95% CI: −0.50 to −0.02; p = 0.03; I² = 0%), respectively. However, this significant advantage was not maintained at longer follow-up intervals (1, 2, and 3 years). The overall pooled analysis indicated a significant improvement in DASH scores in favor of VLP, with an SMD of −0.26 (95% CI: −0.42 to −0.10; p = 0.001). Notably, moderate heterogeneity was present (I² = 55%) (Figure 3).

Grip Strength

**Figure 4:**
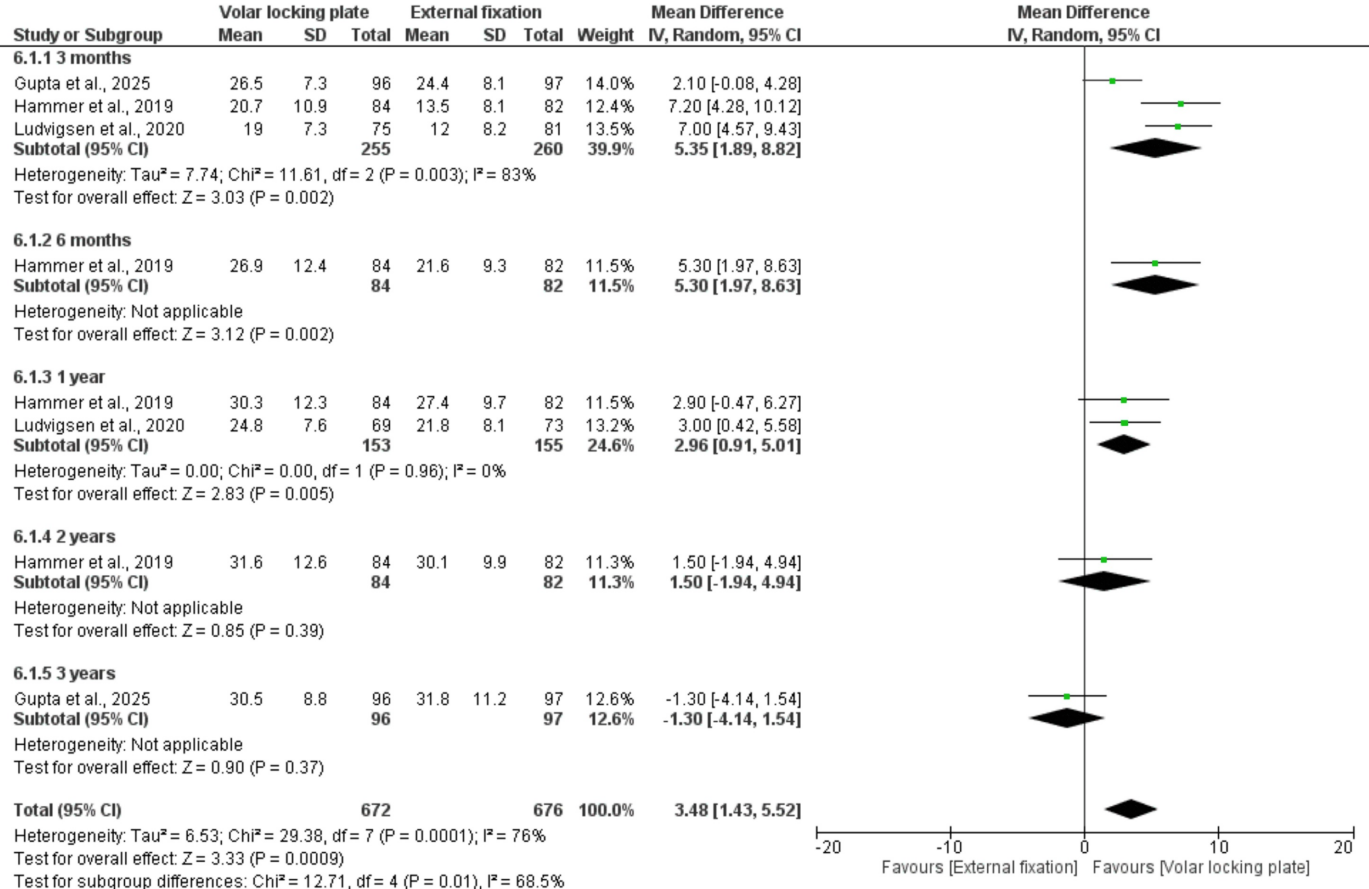
Grip strength Reference [22,26,27].

Three of the included studies reported this outcome. At three months, six months, and one-year post-treatment, patients treated with VLP demonstrated significantly greater grip strength compared with those treated with EF. The mean differences were 5.35 (95% CI: 1.89-8.82; p = 0.002; I² = 83%), 5.30 (95% CI: 1.97-8.63; p = 0.002), and 2.96 (95% CI: 0.91-5.01; p = 0.005; I² = 0%), respectively. However, analysis of grip strength showed no significant difference at two- and three-years post-treatment, the mean differences were 1.50 (95% CI: -1.94-4.94; p = 0.39) and -1.30 (95% CI: -4.14-1.54; p = 0.37), respectively. Overall, the pooled analysis showed a significant improvement in grip strength favoring VLP, with an MD of 3.48 (95% CI: 1.43-5.52; p = 0.0009; I² = 76%) (Figure 4).

VAS Pain Score

**Figure 5:**
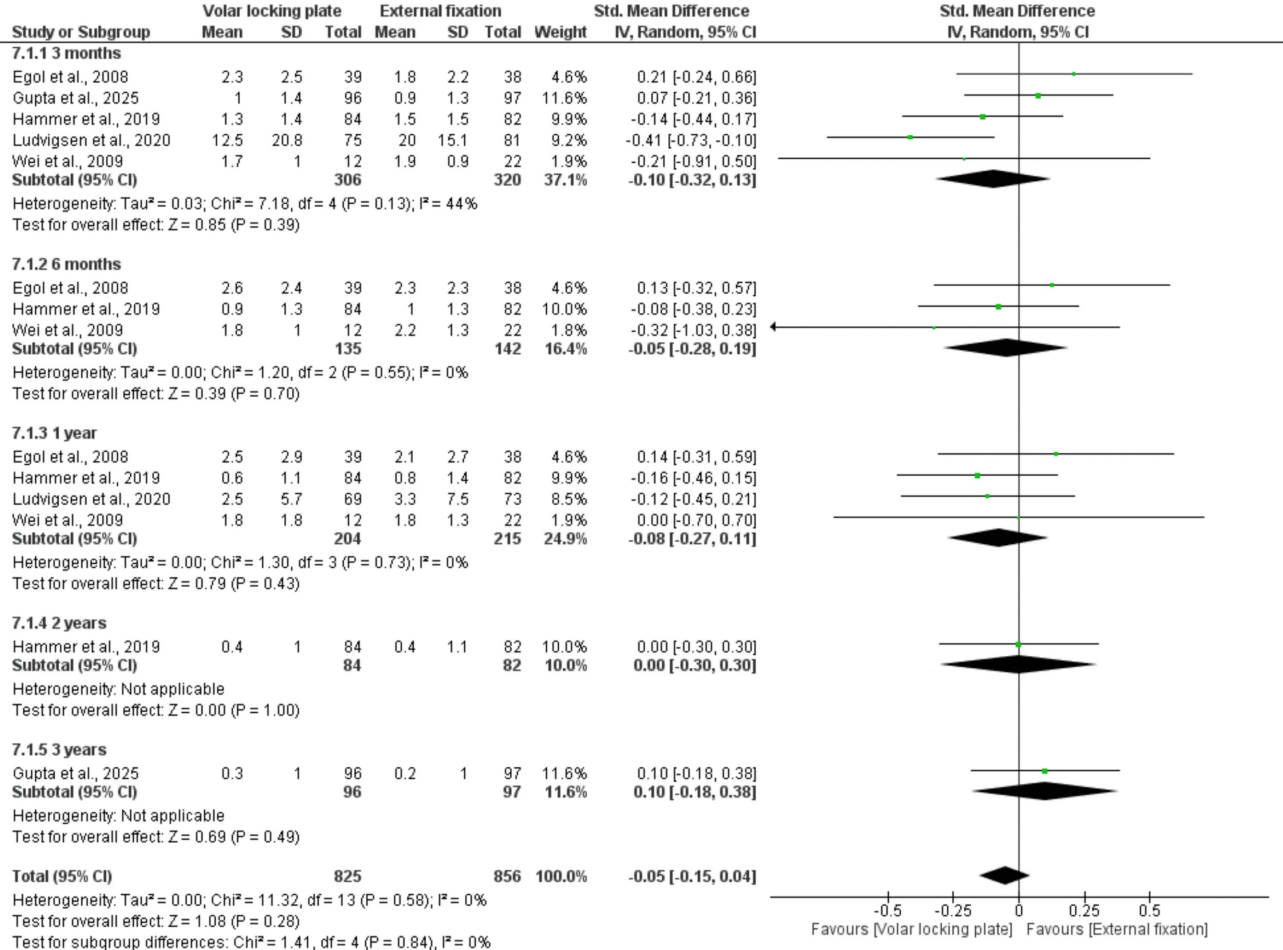
VAS pain score References: [22,23,26-28].

Across all follow-up periods (3 months, 6 months, and 1-3 years), there was no significant difference in pain outcomes between the two treatment groups. The overall pooled analysis also showed no significant advantage of either technique (MD = −0.05; 95% CI: −0.15 to 0.04; p = 0.28) with no heterogeneity (I² = 0%), indicating consistent results across studies (Figure 5).

Secondary outcomes

Wrist Range of Motion (ROM) at Three Months

**Figure 6:**
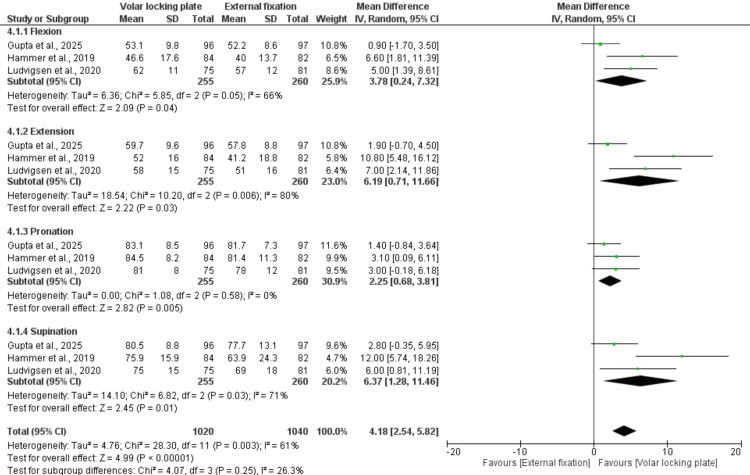
Wrist range of motion (ROM) at three months

At three months post-treatment, pooled data from three studies involving 515 patients showed that VLP provided significantly better wrist ROM than EF across all movement directions. Specifically, VLP demonstrated superior flexion (MD = 3.78; 95% CI: 0.24-7.32; p = 0.04), extension (MD = 6.19; 95% CI: 0.71-11.66; p = 0.03), pronation (MD = 2.25; 95% CI: 0.68-3.81; p = 0.005), and supination (MD = 6.37; 95% CI: 1.28-11.46; p = 0.01). The overall pooled analysis confirmed a significant improvement in total wrist ROM in favor of VLP (MD = 4.18; 95% CI: 2.54-5.82; p < 0.00001). Moderate heterogeneity (I² = 61%) was noted (Figure 6).

Wrist Range of Motion (ROM) at 12 Months

**Figure 7:**
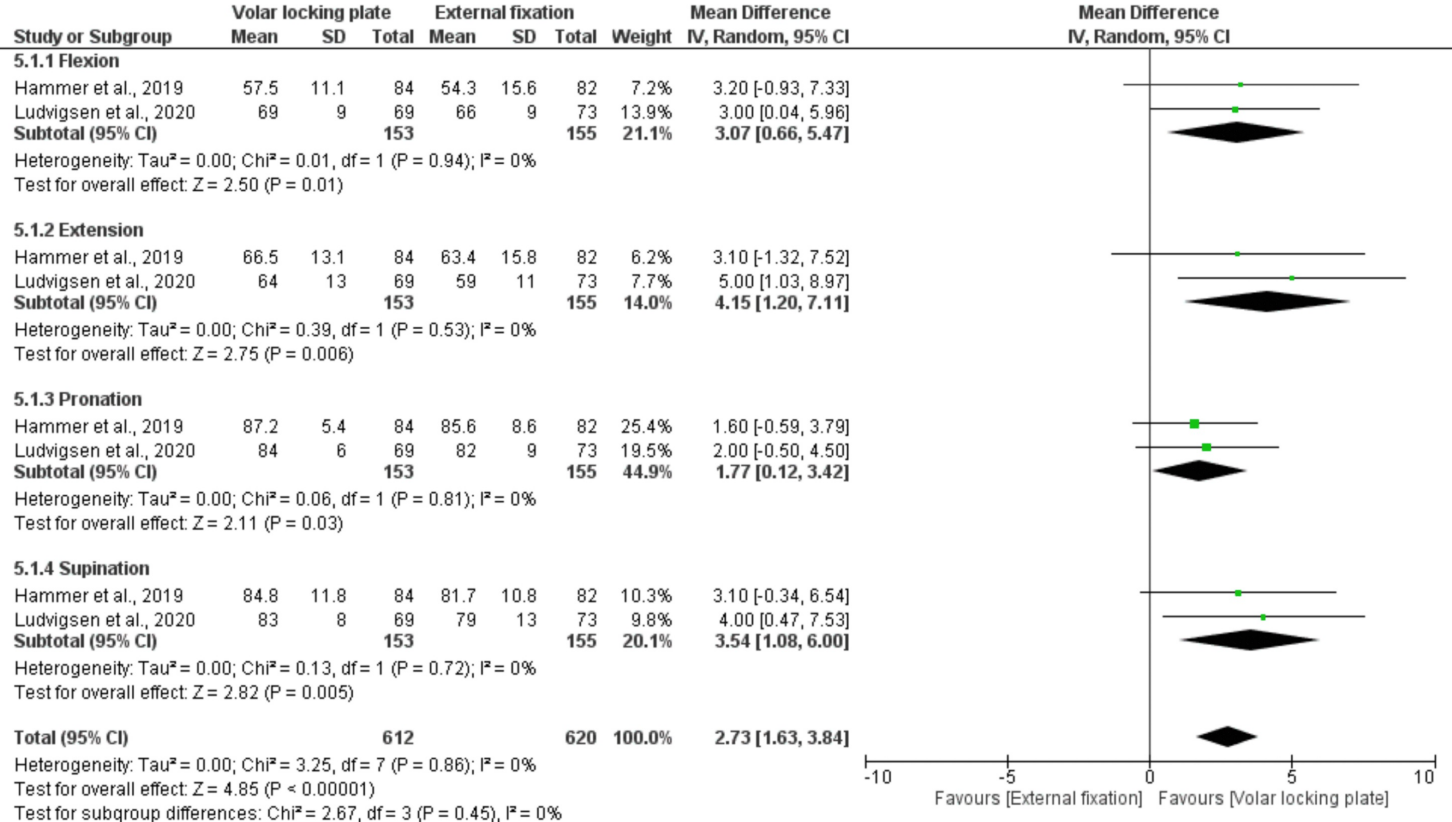
Wrist range of motion (ROM) at 12 months References: [22,27].

Three studies comprising 308 patients evaluated wrist ROM at 12 months and demonstrated a significant advantage of VLP over EF across all movement directions. Specifically, VLP achieved greater flexion (MD = 3.07; 95% CI: 0.66-5.47; p = 0.01), extension (MD = 4.15; 95% CI: 1.20-7.11; p = 0.006), pronation (MD = 1.77; 95% CI: 0.12-3.42; p = 0.03), and supination (MD = 3.54; 95% CI: 1.08-6.00; p = 0.005). The pooled analysis confirmed a significant overall improvement in wrist ROM favoring VLP (MD = 2.73; 95% CI: 1.63-3.84; p < 0.00001) with no observed heterogeneity (I² = 0%) (Figure 7).

Radiological Measurement

**Figure 8:**
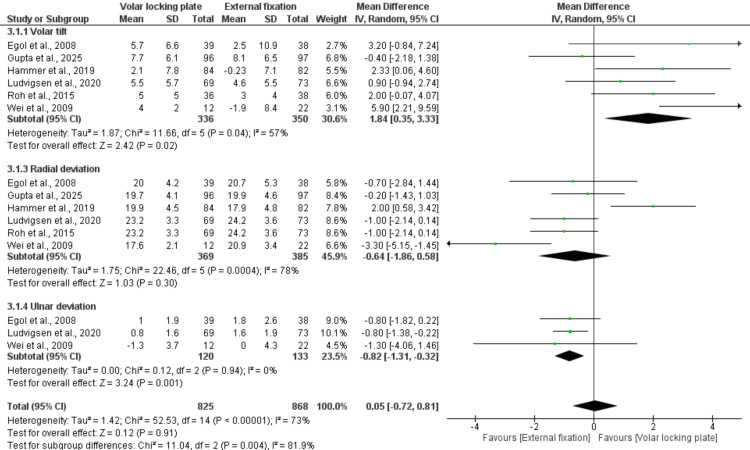
Radiological measurement References: [20,22,23,26-26].

Volar tilt: Six studies involving 686 patients assessed volar tilt and demonstrated a significant improvement in patients treated with VLP compared to those treated with EF (MD = 1.84; 95% CI: 0.35-3.33; p = 0.02). Ulnar deviation: Three studies comprising 253 patients showed a significant advantage of EF over VLP (MD = −0.82; 95% CI: −1.31 to −0.32; p = 0.001). Radial deviation: Five studies, including 754 patients, evaluated radial deviation, showing no significant difference between VLP and EF (MD = −0.64; 95% CI: −1.86 to 0.58; p = 0.30; I² = 78%). The total pooled analysis of radiographic parameters showed no significant difference between VLP and EF (MD = 0.05; 95% CI: −0.72 to 0.81; p = 0.91; I² = 73%). This indicates that both techniques provide comparable radiographic outcomes, although the Significant heterogeneity (I² = 73%) suggests variability among studies (Figure 8).

**Figure 9:**
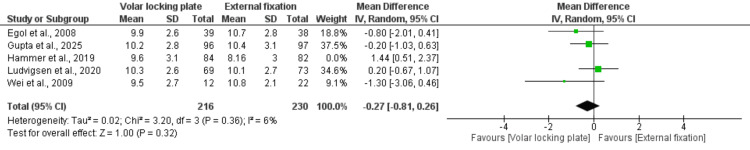
Radial shortening References: [22,23,26-28].

Radial shortening: Five of the included studies, comprising 446 patients, assessed radial shortening and showed no significant difference between VLP and EF in restoring radial length (MD = -0.27; 95% CI: -0.81 to 0.26; p = 0.32). No heterogeneity was observed across studies (I² = 6%) (Figure 9).

Safety outcomes

Minor and Overall Complications

**Figure 10:**
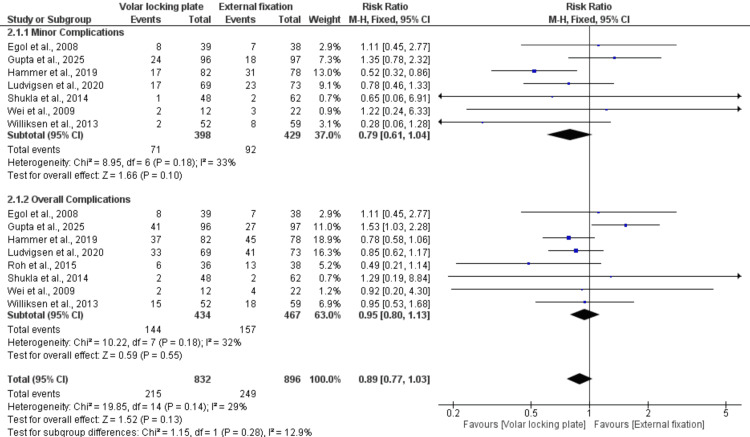
Complications References: [20-24,26-28].

Minor complications: seven studies with 827 patients reported the rate of minor complications and showed no significant difference between VLP and EF (RR = 0.79; 95% CI: 0.61-1.04; p = 0.10). Low heterogeneity was observed across studies (I² = 36%). Overall complications: eight studies, including 901 patients, reported this outcome and demonstrated no significant difference between VLP and EF (RR = 0.95; 95% CI: 0.80-1.13; p = 0.55). Low heterogeneity was observed across studies (I² = 32%). The pooled analysis of all complications showed no significant difference between the two techniques, with comparable total complication rates (RR = 0.89; 95% CI: 0.77-1.03; p = 0.13) and low heterogeneity across studies (I² = 29%) (Figure 10).

**Figure 11:**
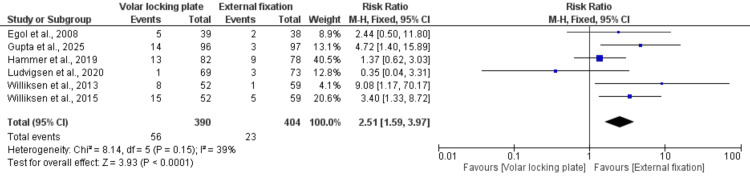
Risk of reoperation References: [22,24-28].

Risk of reoperation: Six of the included studies, comprising 794 patients, reported on the incidence of reoperation. The analysis showed that patients treated with VLP had a significantly higher reoperation rate than those treated with EF (RR = 2.51; 95% CI: 1.59-3.97; p < 0.0001). Low heterogeneity (I² = 39%) was observed (Figure 11).

Discussion

This meta-analysis of nine RCTs involving 1,023 patients demonstrates that VLP provides superior early functional recovery with significantly lower DASH scores at 3 and 6 months, better grip strength up to 12 months, and improved wrist ROM. Pain outcomes, overall complication rates, and long-term functional results at 2-3 years remained comparable between both methods, with no clinically significant differences in radiographic alignment. Clinically, these findings suggest that VLP should be preferred for patients where early functional recovery and durable fixation are priorities. In contrast, EF remains a valid option when a less invasive approach is required.

Previous meta-analyses by Fu et al. and Gouk et al. reported findings consistent with ours, showing that VLP provides better early improvements in pain, grip strength, and ROM than EF [5,29]. However, unlike our results, these studies found that VLP achieved better restoration of ulnar variance, whereas our analysis showed no significant radiological difference between the two techniques, with only minor, clinically insignificant advantages for VLP. Also, consistency was observed in reoperation rates compared with previous reviews. Gouk et al. and Gou et al. reported a higher reoperation rate with VLP, which aligns with our findings [29,30].

Among the included studies in this meta-analysis, studies [20,21,26,27] focused exclusively on intra-articular DRFs. It is a common variant, accounting for approximately 10% to 12% of all DRFs. Furthermore, around 77% of intra-articular DRFs involve the sigmoid notch of the distal radioulnar joint [31,32]. Shukla et al [21] reported superior functional outcomes with EF at 1 year, particularly in patients younger than 50 years. In contrast, Roh et al. and Hammer et al. demonstrated that VLP fixation provided faster functional recovery and better short-term outcomes compared with EF [20,27]. While most trials utilized the DASH or QuickDASH as the primary functional outcome measure, Roh et al. employed the Michigan Hand Questionnaire (MHQ), a valid but distinct patient-reported outcome tool with a different scoring methodology and domain structure [20]. Additionally, Roh et al. did not report pain outcomes using a standardized scale such as the VAS, precluding their inclusion in our pain meta-analysis [20].

Similarly, Shukla et al. utilized the Green and O'Brien scoring system, a composite grading tool that integrates pain, ROM, grip strength, and functional activities into a single categorical outcome [21]. While this approach may have clinical utility for holistic assessment, composite scoring systems inherently limit granular analysis of individual outcome domains. Unlike discrete, independently validated metrics such as VAS for pain, QuickDASH for disability, or standardized dynamometry for grip strength, composite scores cannot be disaggregated for subgroup or sensitivity analyses, reducing their contribution to quantitative synthesis.

In addition to clinical and functional outcomes, economic considerations also play a crucial role in determining the preferred fixation method for DRFs. Karantana et al. (2015) evaluated VLP fixation against EF within the UK NHS and found that VLP was approximately £713 more expensive per patient, offering only minimal improvement in quality-adjusted life years [33]. The resulting incremental cost-effectiveness ratio of £40,068/QALY reached the upper limit of NHS cost-effectiveness, indicating limited economic benefit when only direct healthcare costs are considered. In contrast, Hammer et al. (2020) compared VLP and EF over two years in Norway using both healthcare and societal perspectives. Although healthcare costs and QALYs were similar, EF resulted in more extended work absence (9.2 vs 5.5 weeks), increasing societal costs (€18,037 vs €12,567). Consequently, VLP was 90% more likely to be cost-effective due to reduced productivity loss [34].

Interpretation of reoperation rates is also relevant when assessing the overall clinical and economic impact of fixation strategies. The higher reoperation rate observed with VLP fixation was largely driven by hardware removal, indicating that secondary interventions primarily addressed implant-related complications rather than fixation failure. As VLPs are intended to remain permanently in situ, hardware removal generally reflects the management of complications such as tendon irritation or implant prominence. Accordingly, this finding reflects the distinct complication profile of permanent internal fixation and should not be interpreted as evidence of inferior fracture healing or increased treatment failure.

The strengths of this study include: the inclusion of only RCTs, which enhances the methodological consistency and reliability of the findings. Additionally, the study included a large sample size and a long follow-up period (up to five years), allowing for a more comprehensive evaluation of both early and long-term outcomes. We performed subgroup analysis based on our follow-up duration.

Our study had several limitations. The primary limitation was the high risk of bias, mainly in Domain 4 (outcome measurement), likely due to unblinded assessors and the subjective nature of some outcomes. Additionally, there was variation in fracture types among the included studies; studies focused exclusively on intra-articular DRFs, while others included different fracture patterns, which may have introduced heterogeneity in the results [20,21,26,27].

Future research should focus on larger, high-quality RCTs with long-term follow-up to compare VLP and EF. These trials must reduce bias by using standardized, objective, and blinded outcome measures. Additionally, studies should stratify patients by fracture type and include patient-reported outcomes to identify which technique is best for specific patient groups.

## Conclusions

Our study shows that VLP fixation provides superior early functional recovery and wrist mobility compared with EF. At the same time, pain, radiographic outcomes, and overall complication rates remain comparable between the two techniques. VLP was associated with a higher reoperation rate, whereas EF demonstrated better reoperation outcomes. Both methods achieved similar long-term functional and anatomical outcomes. Therefore, the management of DRFs should be individualized, as no single fixation method demonstrates absolute superiority. VLP is better suited for patients seeking early functional recovery and stable fixation, while EF remains an appropriate choice when a less invasive approach is preferred.

## Appendices

Appendix A

**Table 3:** Full detailed search strategy

Database	Number of Results	Search Strategy
PubMed	719	("Radius"[Mesh] OR "Radius Fractures"[Mesh] OR "Wrist Fractures"[Mesh] OR (distal radius OR distal radial OR colles OR smith) AND (fractur* OR break* OR injury) ) AND ( "Bone Plates"[Mesh] OR "Internal Fixators"[Mesh] OR (volar OR palmar) AND (plate* OR ORIF) OR "locking plate*" OR "locking compression plate*" OR LCP OR VLP OR "internal fixat*" ) AND ( "External Fixators"[Mesh] OR "external fixat*" OR "dynamic external" OR "non-bridging" OR "bridging external" OR "hoffmann II" OR "unilateral frame" )
Web of Science	57	( "Radius" OR "Radius Fractures" OR "Wrist Fractures" OR (distal radius OR distal radial OR colles OR smith) AND (fractur* OR break* OR injury) ) AND ( "Bone Plates" OR "Internal Fixators" OR (volar OR palmar) AND (plate* OR ORIF) OR "locking plate*" OR "locking compression plate*" OR LCP OR VLP OR "internal fixat*" ) AND ( "External Fixators" OR "external fixat*" OR "dynamic external" OR "non-bridging" OR "bridging external" OR "hoffmann II" OR "unilateral frame" )
Scopus	589	( "Radius" OR "Radius Fractures" OR "Wrist Fractures" OR (distal radius OR distal radial OR colles OR smith) AND (fractur* OR break* OR injury) ) AND ( "Bone Plates" OR "Internal Fixators" OR (volar OR palmar) AND (plate* OR ORIF) OR "locking plate*" OR "locking compression plate*" OR LCP OR VLP OR "internal fixat*" ) AND ( "External Fixators" OR "external fixat*" OR "dynamic external" OR "non-bridging" OR "bridging external" OR "hoffmann II" OR "unilateral frame" )
Embase	457	('radius':ab,ti OR 'radius fractures':ab,ti OR 'wrist fractures':ab,ti OR 'distal radius':ab,ti OR 'distal radial':ab,ti OR colles:ab,ti OR smith:ab,ti) AND (fractur*:ab,ti OR break*:ab,ti OR injury:ab,ti) AND (('bone plates':ab,ti OR 'internal fixators':ab,ti OR volar:ab,ti OR palmar:ab,ti) AND (plate*:ab,ti OR orif:ab,ti) OR 'locking plate*':ab,ti OR 'locking compression plate*':ab,ti OR lcp:ab,ti OR vlp:ab,ti OR 'internal fixat*':ab,ti) AND ('external fixators':ab,ti OR 'external fixat*':ab,ti OR 'dynamic external':ab,ti OR 'non-bridging':ab,ti OR 'bridging external':ab,ti OR 'hoffmann ii':ab,ti OR 'unilateral frame':ab,ti)

## References

[1] Nellans KW, Kowalski E, Chung KC (2012). The epidemiology of distal radius fractures. Hand Clin.

[2] Chung KC, Shauver MJ, Birkmeyer JD (2009). Trends in the United States in the treatment of distal radial fractures in the elderly. J Bone Joint Surg Am.

[3] Schneppendahl J, Windolf J, Kaufmann RA (2012). Distal radius fractures: current concepts. J Hand Surg Am.

[4] Forward DP, Davis TR, Sithole JS (2008). Do young patients with malunited fractures of the distal radius inevitably develop symptomatic post-traumatic osteoarthritis?. J Bone Joint Surg Br.

[5] Fu Q, Zhu L, Yang P, Chen A (2018). Volar locking plate versus external fixation for distal radius fractures: A meta-analysis of randomized controlled trials. Indian J Orthop.

[6] Chung KC, Watt AJ, Kotsis SV, Margaliot Z, Haase SC, Kim HM (2006). Treatment of unstable distal radial fractures with the volar locking plating system. J Bone Joint Surg Am.

[7] Macnair RD, Ingham CJ, Davis BJ (2008). Comparison of external and percutaneous pin fixation with plate fixation for intra-articular distal radial fractures. J Bone Joint Surg Am.

[8] Kandemir U, Matityahu A, Desai R, Puttlitz C (2008). Does a volar locking plate provide equivalent stability as a dorsal nonlocking plate in a dorsally comminuted distal radius fracture?: A biomechanical study. J Orthop Trauma.

[9] Nwosu C, Rodriguez K, Zeng S, Klifto KM, Klifto CS, Ruch DS (2023). Complications following volar locking plate fixation of distal radius fractures in adults: A systematic review of randomized control trials. J Hand Surg Am.

[10] Bales JG, Stern PJ (2012). Treatment strategies of distal radius fractures. Hand Clin.

[11] Cumpston M, Li T, Page MJ, Chandler J, Welch VA, Higgins JP, Thomas J (2019). Updated guidance for trusted systematic reviews: A new edition of the Cochrane Handbook for Systematic Reviews of Interventions. Cochrane Database Syst Rev.

[12] Page MJ, McKenzie JE, Bossuyt PM (2021). The PRISMA 2020 statement: An updated guideline for reporting systematic reviews. BMJ.

[13] Sterne JA, Savović J, Page MJ (2019). RoB 2: A revised tool for assessing risk of bias in randomised trials. BMJ.

[14] Hunsaker FG, Cioffi DA, Amadio PC, Wright JG, Caughlin B (2002). The American academy of orthopaedic surgeons outcomes instruments: normative values from the general population. JBJS.

[15] Beaton DE, Wright JG, Katz JN (2005). Development of the QuickDASH: comparison of three item-reduction approaches. J Bone Joint Surg Am.

[16] Katz J, Melzack R (1999). Measurement of pain. Surg Clin North Am.

[17] Bechtol CO (1954). Grip test; the use of a dynamometer with adjustable handle spacings. J Bone Joint Surg Am.

[18] Incel NA, Ceceli E, Durukan PB, Erdem HR, Yorgancioglu ZR (2002). Grip strength: Effect of hand dominance. Singapore Med J.

[19] Medoff RJ (2005). Essential radiographic evaluation for distal radius fractures. Hand Clin.

[20] Roh YH, Lee BK, Baek JR, Noh JH, Gong HS, Baek GH (2015). A randomized comparison of volar plate and external fixation for intra-articular distal radius fractures. J Hand Surg Am.

[21] Shukla R, Jain RK, Sharma NK, Kumar R (2014). External fixation versus volar locking plate for displaced intra-articular distal radius fractures: A prospective randomized comparative study of the functional outcomes. J Orthop Traumatol.

[22] Ludvigsen T, Matre K, Gudmundsdottir RS, Krukhaug Y, Dybvik EH, Fevang JM (2021 ). Surgical treatment of distal radial fractures with external fixation versus volar locking plate: A multicenter randomized controlled trial. J Bone Joint Surg Am.

[23] Wei DH, Raizman NM, Bottino CJ, Jobin CM, Strauch RJ, Rosenwasser MP (2009). Unstable distal radial fractures treated with external fixation, a radial column plate, or a volar plate. A prospective randomized trial. J Bone Joint Surg Am.

[24] Williksen JH, Frihagen F, Hellund JC, Kvernmo HD, Husby T (2013). Volar locking plates versus external fixation and adjuvant pin fixation in unstable distal radius fractures: A randomized, controlled study. J Hand Surg Am.

[25] Williksen JH, Husby T, Hellund JC, Kvernmo HD, Rosales C, Frihagen F (2015). External fixation and adjuvant pins versus volar locking plate fixation in unstable distal radius fractures: A randomized, controlled study with a 5-year follow-up. J Hand Surg Am.

[26] Gupta M, Goyal A, Rohela R, Pal CP (2025). External versus internal fixation of intra-articular distal radius fractures: A randomised controlled trial. Acta Orthop Belg.

[27] Hammer OL, Clementsen S, Hast J, Šaltytė Benth J, Madsen JE, Randsborg PH (2019). Volar locking plates versus augmented external fixation of intra-articular distal radial fractures: Functional results from a randomized controlled trial. J Bone Joint Surg Am.

[28] Egol K, Walsh M, Tejwani N, McLaurin T, Wynn C, Paksima N (2008). Bridging external fixation and supplementary Kirschner-wire fixation versus volar locked plating for unstable fractures of the distal radius: A randomised, prospective trial. J Bone Joint Surg Br.

[29] Gouk CJ, Bindra RR, Tarrant DJ, Thomas MJ (2018). Volar locking plate fixation versus external fixation of distal radius fractures: A meta-analysis. J Hand Surg Eur Vol.

[30] Gou Q, Xiong X, Cao D, He Y, Li X (2021). Volar locking plate versus external fixation for unstable distal radius fractures: A systematic review and meta-analysis based on randomized controlled trials. BMC Musculoskelet Disord.

[31] Turner RG, Faber KJ, Athwal GS (2007). Complications of distal radius fractures. Orthop Clin North Am.

[32] Tanabe K, Nakajima T, Sogo E, Denno K, Horiki M, Nakagawa R (2011). Intra-articular fractures of the distal radius evaluated by computed tomography. J Hand Surg Am.

[33] Karantana A, Scammell BE, Davis TR, Whynes DK (2015). Cost-effectiveness of volar locking plate versus percutaneous fixation for distal radial fractures: Economic evaluation alongside a randomised clinical trial. Bone Joint J.

[34] Hammer OL, Jakobsen RB, Clementsen S, Fuglesang H, Bjornelv GW, Randsborg PH (2020). Cost-effectiveness of volar Locking plate compared with augmented external fixation for displaced intra-articular wrist fractures. J Bone Joint Surg Am.

